# Viral suppression among children and their caregivers living with HIV in western Kenya

**DOI:** 10.1002/jia2.25272

**Published:** 2019-04-15

**Authors:** John M Humphrey, Becky L Genberg, Alfred Keter, Beverly Musick, Edith Apondi, Adrian Gardner, Joseph W Hogan, Kara Wools‐Kaloustian

**Affiliations:** ^1^ Department of Medicine Indiana University Indianapolis IN USA; ^2^ Department of Epidemiology Johns Hopkins Bloomberg School of Public Health Baltimore MD USA; ^3^ Academic Model Providing Access to Healthcare (AMPATH) Eldoret Kenya; ^4^ Department of Biostatistics Indiana University Indianapolis IN USA; ^5^ Department of Paediatrics Moi Teaching and Referral Hospital Eldoret Kenya; ^6^ Department of Biostatistics Brown University Providence RI USA

**Keywords:** HIV, child, caregiver, viraemia, adherence, sub‐Saharan Africa

## Abstract

**Introduction:**

Despite the central role of caregivers in managing HIV treatment for children living with HIV, viral suppression within caregiver–child dyads in which both members are living with HIV is not well described.

**Methods:**

We conducted a retrospective analysis of children living with HIV <15 years of age and their caregivers living with HIV attending HIV clinics affiliated with the Academic Model Providing Access to Healthcare (AMPATH) in Kenya between 2015 and 2017. To be included in the analysis, children and caregivers must have had ≥1 viral load (VL) during the study period while receiving antiretroviral therapy (ART) for ≥6 months, and the date of the caregiver's VL must have occurred ±90 days from the date of the child's VL. The characteristics of children, caregivers and dyads were descriptively summarized. Multivariable logistic regression was used to estimate the odds of viral non‐suppression (≥ 1000 copies/mL) in children, adjusting for caregiver and child characteristics.

**Results:**

Of 7667 children who received care at AMPATH during the study period, 1698 were linked to a caregiver living with HIV and included as caregiver–child dyads. For caregivers, 94% were mothers, median age at ART initiation 32.8 years, median CD4 count at ART initiation 164 cells/mm^3^ and 23% were not virally suppressed. For children, 52% were female, median age at ART initiation 4.2 years, median CD4 values at ART initiation were 15% (age < 5 years) and 396 cells/mm^3^ (age ≥ 5 years), and 38% were not virally suppressed. In the multivariable model, children were found more likely to not be virally suppressed if their caregivers were not suppressed compared to children with suppressed caregivers (aOR = 2.40, 95% CI: 1.86 to 3.10). Other characteristics associated with child viral non‐suppression included caregiver ART regimen change prior to the VL, caregiver receipt of a non‐NNRTI‐based regimen at the time of the VL, younger child age at ART initiation and child tuberculosis treatment at the time of the VL.

**Conclusions:**

Children were at higher risk of viral non‐suppression if their caregivers were not virally suppressed compared to children with suppressed caregivers. A child's viral suppression status should be closely monitored if his or her caregiver is not suppressed.

**Abbreviations**
AMPATH: Academic Model Providing Access to Healthcare
aOR: adjusted odds ratio
ART: antiretroviral therapy
ARV: antiretroviral
CDC: Centers for Disease Control
CI: confidence interval
II: integrase inhibitor
IQR: interquartile range
MTRH: Moi Teaching and Referral Hospital
NNRTI: non‐nucleoside reverse transcriptase inhibitor
PI: protease inhibitor
TB: tuberculosis
VL: viral load
WHO: World Health Organization

## 1 Introduction

The scale‐up of antiretroviral therapy (ART) in sub‐Saharan Africa has improved survival of both children living with HIV and their parents and caregivers living with HIV 1. In these relationships, caregivers must assume responsibility for their own HIV management as well as their child's management. However, both individuals may be vulnerable to medical, economic and psychosocial stressors that may act as barriers to maintaining viral suppression and health for each individual and the relationship as a whole, suggesting the potential importance of family‐centred HIV services for these caregiver–child dyads 2, 3. Yet, despite the central role of caregivers in the management of children living with HIV and the common barriers to viral suppression they may both encounter, the association between viral non‐suppression in caregiver–child dyads is not well understood.

Various studies describing caregiver–child interactions in the context of HIV, particularly in resource‐limited settings, suggest the possibility that viral suppression in children and their caregivers is associated. Poverty, substance abuse, physical and mental illness, and perceived lack of social support may jeopardize adherence to ART for both caregivers and children 2, 4. Having a caregiver other than the mother, experiencing a change in caregiver, and not having a caregiver attend a child's clinic appointment have been associated with lower adherence and higher odds of virologic failure among children and adolescents 5, 6, 7, 8. A mother's attitude and behaviour regarding her own ART adherence may also influence her adherence practices towards her child 9. Such influences could be positive (e.g. modelling good adherence and beliefs about ART efficacy) or negative (e.g. externalizing feelings of stigma and guilt, concerns over ART side effects, forgetting ART doses or medication fatigue), and dynamic over time as children gain independence and family structures evolve 10, 11. Children may miss ART doses if their caregivers are unavailable or struggling to manage their own HIV infection and associated conditions 2, 12. HIV‐related stigma and disclosure concerns may further inhibit caregivers from administering ART to their children or bringing them to clinic 13, 14, 15, 16, 17.

HIV viral load (VL) testing has recently been introduced in Kenya and other countries in sub‐Saharan Africa for routine treatment monitoring 18, 19, 20. This presents a novel opportunity to examine caregiver–child viral suppression in this region. Given the risk of HIV drug resistance and HIV disease progression in the setting of HIV viraemia, characterizing the drivers of viral non‐suppression in children is important 21. The objective of this study is to describe the association between child and caregiver viral suppression and the factors that influence viral non‐suppression in children.

## 2 Methods

### 2.1 Study design

This retrospective cohort study used electronic medical record data from children living with HIV and their adult caregivers living with HIV who received HIV care at the Academic Model Providing Access to Healthcare (AMPATH) programme in western Kenya between 2015 and 2017. This study was approved by the Institutional Research and Ethics Committee at Moi University in Kenya and Indiana University IRB. Patient‐level consent was waived by the regulatory bodies because the data were collected as part of routine care and were de‐identified prior to analysis.

### 2.2 Study setting and population

AMPATH is a USAID‐funded HIV care and treatment programme situated in a generalized HIV epidemic setting in western Kenya 22. AMPATH is a participating site in the International Epidemiology Databases to Evaluate AIDS East Africa consortium 23. The county‐level HIV prevalence in the AMPATH catchment ranges from 1.6% to 20.7% among adults ≥15 years of age. In these counties, there are an estimated 446,693 HIV‐positive adults ≥15 years of age and 36,743 HIV‐positive children 0 to 14 years of age, with ART coverage ranging from 47% to 100% for adults and 49% to 98% for children as of 2017 24. Since 2001, AMPATH has enrolled over 150,000 patients living with HIV at Ministry of Health facilities across western Kenya and currently provides HIV care to approximately 85,000 patients, including over 7500 children 25, 26. All facilities provide standard of care HIV treatment services based on national guidelines, which in 2015 recommended ART initiation for all children ≤10 years of age and adolescents and adults with CD4 count <500 cells/mm^3^ 19. The WHO Option B+ policy recommending lifelong ART for all pregnant women living with HIV was adopted by the Kenya Ministry of Health and AMPATH in 2014 19. In 2016, AMPATH transitioned to universal ART eligibility 27. Recommended first‐line ART at the time of the study was lopinavir‐based for children <3 years of age and efavirenz‐based for children three to fourteen years of age and adults ≥15 years of age.

Within the AMPATH programme there is a dedicated paediatric HIV clinic at Moi Teaching and Referral Hospital with paediatric‐dedicated clinical officers and paediatricians. However, at all other facilities, the same clinical officers treat both children and adults either in separate or combined adult/paediatric clinics. The study population included all children living with HIV and their caregivers living with HIV who attended an AMPATH affiliated HIV clinic at any time from 1 January 2015 to 14 February 2017. Routine VL monitoring was implemented for all patients at AMPATH during this period, replacing the previous recommendation for immunologic monitoring. At the time of the study, WHO and Kenyan HIV treatment guidelines recommended VL testing six months after ART initiation, and if ≤1000 copies/mL, annually thereafter 27, 28. Individuals with a VL ≥1000 copies/mL were recommended to have the VL test repeated after a minimum of three months of enhanced adherence counselling and support. CD4 testing was recommended at baseline, but not routinely thereafter, for individuals on ART with access to VL testing 19.

Caregiver–child dyads were selected for the study according to the following criteria: First, children were included in the study if they were: (1) <15 years of age on or after 1 January 2015 (study start date); (2) living with HIV and enrolled in care at AMPATH; (3) receiving ART for at least six months prior to 14 February 2017 (database closure); and (4) documented to have at least one VL measure while receiving ART for at least six months. We then excluded all children who were not linked to any caregiver in the medical record. Caregiver was defined as any individual living with HIV and categorized as mother, father, aunt, uncle, grandparent, stepparent, foster parent, guardian or caretaker. Although siblings could also act as caregivers in the Kenya context (e.g. for orphaned children whose parent(s) had died of HIV), the medical record did not contain information to substantiate siblings’ status as caregivers so were not included in the caregiver definition 29. Subsequently, we included caregivers according to the above criteria 2 to 4 used for children, along with the additional criterion that each caregiver has at least one VL measure ±90 days from the date of the child's VL measure during the study period. In the event that there was more than one eligible VL pair for a given dyad during the study period, we sampled the first chronological caregiver–child VL pair to serve as the unit of analysis for each individual. For caregivers linked to more than one child, we included each child in the analysis so that, for example, a caregiver linked to two children would be considered two caregiver–child dyads with the caregiver counted twice. For children linked to multiple caregivers, the mother was preferentially selected, followed by the father. In western Kenya and other contexts in sub‐Saharan Africa, mothers are commonly the primary caregivers for children living with HIV 5, 30, 31. We elected to restrict our sample to those caregiver–child VLs that occurred within 90 days of one another, as we assumed this to be a reasonable period in which a temporal association between caregiver–child viral suppression could be evaluated. Our decision to include VL measures that occur at least six months after initiating ART is also consistent with World Health Organization (WHO) and Kenyan HIV treatment guidelines, as elevated VL measures within six months may represent normal values along the continuum of VL decline for patients with adequate adherence after initiating ART 27, 32. Finally, we included children that were <15 years of age because age ≥15 is an established cutoff used to define “young adults” or “adults” according to the Kenyan Ministry of Health and other international health agencies 27, 33, 34, 35.

### 2.3 Data management

We used clinical and viral load data available in the AMPATH electronic medical record (EMR) that was collected during routine care initially on paper‐based forms and from 2016 by point‐of‐care data entry 36. The demographic section of the EMR contains a “relationships” function that enables the user to manually enter the names and medical record numbers of other AMPATH patients (e.g. spouses, children, other family members/caregivers) and designate the type of relationship the linkage represents (e.g. parent/child, sibling/sibling, grandparent/grandchild, etc.). These relationship links are recorded in the EMR by clinicians as part of routine clinical documentation. We used this linkage data to identify the caregiver–child dyads in our study. These data were de‐identified prior to analysis.

### 2.4 Statistical analysis

The primary outcome measure was viral non‐suppression among children, defined as ≥1000 copies/mL. The following independent variables were included for caregivers and children: *Enrolment in HIV care* – sex, mother/father vital status, caregiver type, total number of children each caregiver has (includes HIV‐positive children included and excluded from the study, as well as children who are HIV‐negative); *ART initiation* – age, WHO stage/CDC class, CD4 count/per cent; *Characteristics during the study period* – proportion of caregivers that attended same clinic as their linked children, total number of VL measures available for each individual, facility type (Moi Teaching and Referral Hospital vs. other); *Characteristics at the time of the VL pair selected for analysis* – age, pregnancy status (caregiver only), tuberculosis (TB) treatment status, number of days between caregiver and child VLs selected for analysis, antiretroviral (ARV) base class, ART line (according to Kenyan national treatment guidelines 27), whether an ARV base class switch occurred due to treatment failure at any time prior to the VL during the study period, and whether an ART regimen change occurred for any reason (except dose changes) at any time prior to the VL during the study period.

Logistic regression model fitted using generalized estimating equations (GEE) was used to calculate unadjusted odds ratios (OR) and 95% confidence intervals (CI) for independent variables to assess their associations with viral non‐suppression in children. A multivariable logistic regression model fitted using the same approach was then constructed to estimate the adjusted association between child viral non‐suppression and caregiver and child characteristics that were significant in the bivariable model 37, 38, 39. The bivariable and multivariable models were set‐up so that the child viral suppression status was the dependent variable and the caregiver suppression status was the independent variable. The effect of the caregiver suppression status was adjusted for the following caregiver and child characteristics: sex, age (years) at ART initiation, time (years) on ART, being on TB treatment at the time of the VL, WHO stage/CDC class at ART initiation, CD4 count or % at ART initiation, NNRTI base class at the time of the VL, ART regimen change for any reason before the VL, VL not suppressed, type of caregiver, number of children at the time of the VL (caregiver only), pregnant at the time of the VL (caregiver only), MTRH facility, caregiver vital status (child only) and the interaction between age at ART initiation and time on ART. GEE using exchangeable correlation structure and robust variance estimation was used to handle the effect of clustering of children within caregiver units in the model.

## 3 Results

**Figure 1:**
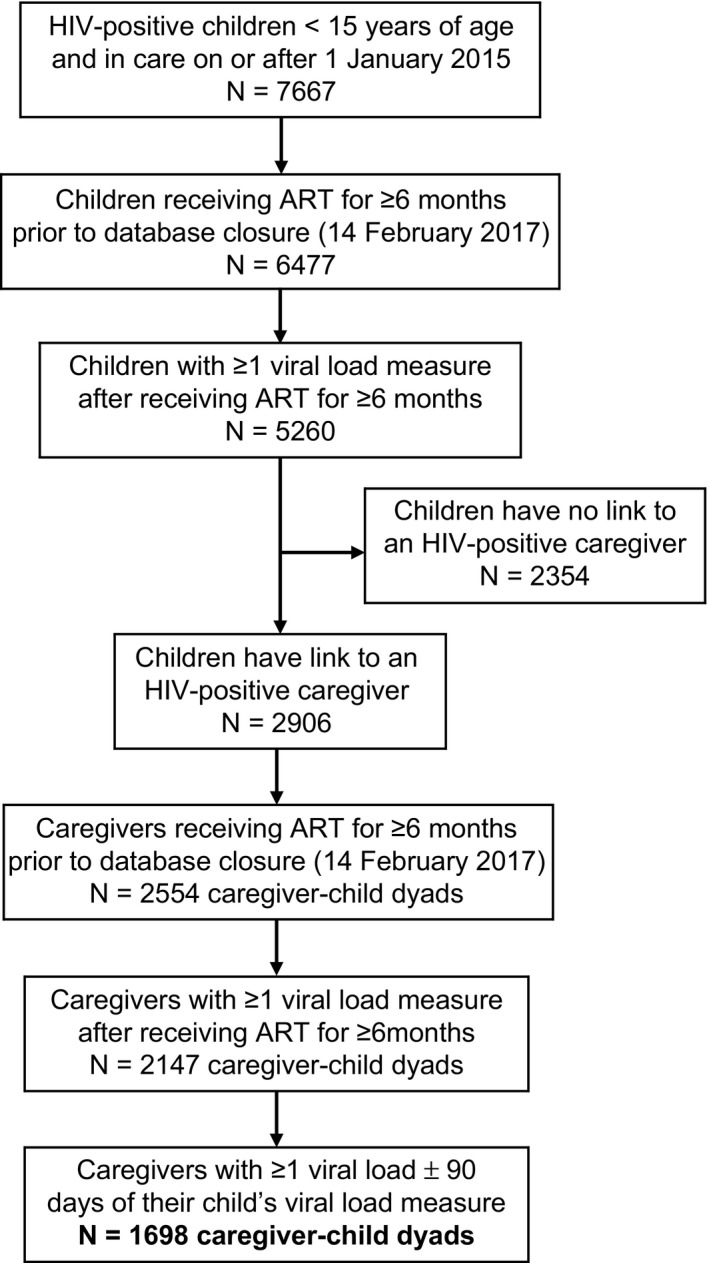
Caregiver–child dyad selection

A total of 7667 children living with HIV received care at an AMPATH clinic at least once during the study period (Figure 1). Of these, 5260 met the inclusion criteria, and of those, 2906 were linked to a caregiver living with HIV who also received care at an AMPATH clinic during the study period. After eliminating linked caregivers who did not meet the inclusion criteria (37% of caregivers were eliminated because they did not have a VL ±90 days of the child VL), a total of 1698 caregiver–child dyads were eligible for the analysis. The age at ART initiation and proportion of females were similar between children included in the analysis and all children excluded from the analysis, as well as among the subset of children excluded because they did not have a link to a caregiver living with HIV. The distribution of dyad types was: mother–daughter (49%), mother–son (45%), father–son (3%) and father–daughter (2%).

### 3.1 Caregiver characteristics

There were 1639 unique caregivers included in the analysis, among whom 94% were mothers (Table 1). At enrolment, 3% (n = 54) of caregivers were linked to more than one child living with HIV, with 50 linked to two children, three linked to three children, and one linked to four children. Caregivers had a median of three children in total (with and without HIV). At ART initiation, the median caregiver age was 32.8 years. Caregivers’ median CD4 count (interquartile range [IQR]) at ART initiation was 164 (86 to 258) cells/mm^3^ among those with available data and the majority (88%) had a CD4 count ≤350 cells/mm^3^. Among caregivers with available WHO stage at ART initiation, 36% had Stage 3 or 4 disease. During the study period, caregivers attended the same clinic as their linked child in 99% of cases, with the caregiver bringing the child to clinic at least once during the study period in 94% of cases. Prior to the date of the caregiver VL during the study period, less than 1% of caregivers experienced an ARV base class switch due to treatment failure, while 8% experienced an ART regimen change for any reason. At the time of the caregiver VL, caregivers’ median age was 38.6 years, 0.7% were pregnant and 0.7% were on TB treatment. The median time on ART at the time of the VL was 5.7 years. A total of 89% of caregivers were receiving non‐nucleoside reverse transcriptase inhibitor (NNRTI)‐based ART and 89% of ART regimens were categorized as first line. The VL was not suppressed in 23% of caregivers.

**Table 1:** Characteristics of caregivers

Characteristic	N = 1639, n (%)
Female	1551 (95%)
Age at ART initiation, median years (IQR)	32.8 (28.5 to 37.2)
Age at VL, median years (IQR)	38.6 (33.7 to 43.3)
Time on ART, median years (IQR)	5.7 (3.6 to 7.9)
Type of caregiver	
Mother	1535 (94%)
Father	81 (4.9%)
Other^a^	23 (1.4%)
Number of children at enrolment, median (IQR)	3 (2 to 5)
Pregnant at VL^b^	11 (0.7%)
On TB treatment at VL	11 (0.7%)
Facility	
MTRH	333 (20%)
Other	1306 (80%)
WHO stage at ART initiation	
Stage 1 or 2	985 (60%)
Stage 3 or 4	550 (34%)
Missing	104 (6.4%)
CD4 count at ART initiation, median (IQR)	164 (86 to 258)
≤350	875 (53%)
>350	119 (7.3%)
Missing	645 (39%)
ARV base class at VL	
NNRTI	1459 (89%)
PI	178 (11%)
Other (i.e. PI + NNRTI or PI + II)	2 (0.1%)
ART line at VL	
First line	1461 (89%)
Second line	177 (11%)
Third line	1 (0.1%)
ARV base class switch for treatment failure before VL^c^	7 (0.4%)
ART regimen change for any reason before VL^d^	134 (8.2%)
VL not suppressed	376 (23%)

ART, antiretroviral therapy; ARV, antiretroviral; II, integrase inhibitor; IQR, interquartile range; MTRH, Moi Teaching and Referral Hospital; NNRTI, non‐nucleoside reverse transcriptase inhibitor; PI, protease inhibitor; TB, tuberculosis; VL, viral load; WHO, World Health Organization.

^a^Other caregiver types include: step‐parent (n = 16), guardian (n = 5), grandparent (n = 1), uncle (n = 1); ^b^proportion expressed among female caregivers only (n = 1551); ^c^indicates any ART switch due to failure from first‐ to second‐line or from second‐ to third‐line before the VL date according to Kenya HIV treatment guidelines; ^d^excludes dose change.

### 3.2 Child characteristics

For children, 52% of 1698 were female (Table 2). At ART initiation, the median child age was four years and 93% were <10 years of age, and 3% (n = 52) were linked to more than one caregiver. Among children with available parental vital status data at enrolment, the mother was deceased in 6% and father was deceased in 27%. At ART initiation, the WHO stage/CDC class was Stage 3 or 4/class B or C in 46% of children with available data. Among children <5 years of age, the CD4% at ART initiation was 15% (IQR 10 to 22); for children ≥5 years of age, the median CD4 count was 396 cells/mm^3^ (IQR 234 to 686). Two‐thirds of children with available data had a CD4% >25% (<5 years) or >350 cells/mm^3^ (>5 years) at ART initiation. Prior to the date of the child VL, 0.8% of children experienced an ARV base class switch due to treatment failure and 5.2% experienced an ART regimen change for any reason. At the time of the child VL used in the analysis, the median child age was 9.7 years and 0.8% were on TB treatment. The median time on ART for children at the time of the VL ranged from 1.2 years for children 0 to 2 years of age to 5.7 years for children 10 to 14 years of age. A total of 94% of children were receiving NNRTI‐based ART and 96% of children were categorized as receiving first‐line ART. The VL was not suppressed in 38% of children. Among children on ART ≥6 months and with ≥1 VL available measure who were excluded from the analysis because they did not have a link to an HIV‐positive caregiver (n = 2354; see Figure 1), the VL was not suppressed in 33% (n = 1576; *p *< 0.001 compared to children included in the analysis). Additionally, viral suppression among excluded children with documented orphan status (n = 1439, defined as mother or both parents deceased and using the earliest VL measure available during the study period) was 68%, compared to 64% for children without documented orphan status (n = 885) (*p *=* *0.04).

**Table 2:** Characteristics of children

Characteristic	N = 1698, n (%)
Female	885 (52%)
Age at ART initiation	
0 to 2 years	621 (36%)
3 to 5 years	502 (30%)
6 to 9 years	463 (27%)
10 to 14 years	112 (6.6%)
Age at VL	
0 to 2 years	85 (5.0%)
3 to 5 years	236 (14%)
6 to 9 years	586 (34%)
10 to 14 years	791 (47%)
Time on ART, median years (IQR)	
0 to 2 years	1.2 (0.8 to 1.7)
3 to 5 years	3.0 (1.9 to 4.0)
6 to 9 years	4.9 (2.8 to 6.1)
10 to 14 years	5.7 (3.7 to 7.6)
Caregiver vital status	
Mother deceased (n = 1093 with available data)	68 (6.2%)
Father deceased (n = 1063 with available data)	288 (27%)
Mother or father deceased (n = 1189 with available data)	326 (27%)
On TB treatment at VL	14 (0.8%)
WHO stage/CDC class at ART initiation	
Stage 1 or 2/Class N or A	700 (41%)
Stage 3 or 4/Class B or C	602 (36%)
Missing	396 (23%)
CD4 % at ART initiation for children <5 years, median (IQR) (n = 450 with available data)	15 (10 to 22)
CD4 count at ART initiation for children ≥5 years, median (IQR) (n = 349 with available data)	396 (234 to 686)
CD4 % at ART initiation >25 for children <5 years|CD4 count at ART initiation >350 for children aged ≥5 years	
No	519 (31%)
Yes	280 (17%)
Missing	899 (53%)
ARV base class at VL	
NNRTI	1590 (94%)
PI	108 (6.4%)
ART line at VL	
First line	1626 (96%)
Second line	72 (4.2%)
ARV base class switch for treatment failure before VL^a^	14 (0.8%)
ARV regimen change for any reason before VL^b^	89 (5.2%)
VL not suppressed	652 (38%)

ART, antiretroviral therapy; ARV, antiretroviral; CDC, Centers for Disease Control; II, integrase inhibitor; NNRTI, non‐nucleoside reverse transcriptase inhibitor; PI, protease inhibitor; TB, tuberculosis; VL, viral load; WHO, World Health Organization.

^a^Indicates any ART switch from first‐ to second‐line or from second‐ to third‐line before the VL date according to Kenya HIV treatment guidelines; ^b^excludes dose changes.

### 3.3 Viral load characteristics

There was a median (IQR) of 2 (2 to 3) available VL measures each for caregivers and children during the study period. The median (IQR) number of days between the caregiver and child VL measures used for analysis was 5 (0 to 55) days out of a maximum of 90 days according to the inclusion criteria. The viral suppression status (<1000 copies/mL) for all 1698 caregiver–child dyads was: caregiver and child both suppressed (n = 880 dyads, 52%), caregiver suppressed and child not suppressed (n = 430 dyads, 25%), caregiver not suppressed and child suppressed (n = 166 dyads, 10%), and caregiver and child both not suppressed (n = 222 dyads, 13%).

### 3.4 Associations with viral non‐suppression in children

In Table 3, caregiver and child characteristics that were significantly associated (*p *≤* *0.05) with child viral non‐suppression in the bivariable model were included in the multivariable model. The sex of the caregiver and child was also included in the multivariable model despite not being statistically significant in the bivariable model given previously observed sex differences in HIV treatment outcomes 40, 41. Children with caregivers who were not virally suppressed were more than twice as likely to not be virally suppressed themselves compared to children with suppressed caregivers (adjusted odds ratio [aOR]=2.40, 95% CI: 1.86 to 3.10; Table 3). Characteristics associated with a higher adjusted odds of viral non‐suppression in children included caregiver ART regimen change for any reason before the VL (aOR = 1.82, 95% CI: 1.21 to 2.72), the child being on TB treatment at the time of the VL (aOR = 3.32, 95% CI: 1.13 to 9.81). Characteristics associated with a lower adjusted odds of viral non‐suppression in children included caregiver receipt of an NNRTI‐based regimen at the time of the VL (aOR = 0.69, 95% CI 0.50 to 0.97), and child age six to nine years (aOR = 0.49, 95% CI 0.27 to 0.89) and 10 to 14 years (aOR = 0.30, 95% CI 0.11 to 0.82) compared to zero to two years.

**Table 3:** Unadjusted and adjusted odds ratios for factors associated with viral non‐suppression in children

Characteristic	N	Unadjusted OR (95% CI)	Adjusted OR (95% CI) N* *= 1608
Caregiver characteristics			
Female	1639	1.14 (0.73 to 1.78)	0.84 (0.52 to 1.36)
Age (years) at ART initiation	1635	0.97 (0.96 to 0.99)	0.98 (0.96 to 1.00)
Time (years) on ART	1635	1.00 (0.97 to 1.04)	0.99 (0.94 to 1.04)
Type of caregiver			
Mother versus other	1639	0.82 (0.36 to 1.87)	
Father versus other		0.66 (0.26 to 1.70)	
Number of children at VL	1504	0.98 (0.93 to 1.04)	
Pregnant at VL	1551	2.80 (0.82 to 9.63)	
On TB treatment at VL	1639	2.83 (0.82 to 9.69)	1.70 (0.49 to 5.84)
MTRH facility	1639	1.00 (0.78 to 1.28)	
WHO Stage 3 to 4 at ART initiation			
Stage 3 or 4 versus Stage 1 or 2	1639	0.93 (0.75 to 1.15)	
Missing versus Stage 1 or 2		1.05 (0.70 to 1.58)	
CD4 count at ART initiation >350			
Yes versus no	1639	0.99 (0.67 to 1.46)	1.23 (0.81 to 1.86)
Missing versus no		1.21 (0.98 to 1.49)	1.17 (0.94 to 1.47)
NNRTI base class at VL (vs. PI/other regimen)	1639	0.65 (0.47 to 0.88)	0.69 (0.50 to 0.97)
ART regimen change for any reason before VL	1639	2.89 (2.02 to 4.13)	1.82 (1.21 to 2.72)
VL not suppressed	1639	2.72 (2.15 to 3.43)	2.40 (1.86 to 3.10)
Children characteristics			
Female	1639	0.89 (0.73 to 1.08)	0.86 (0.70 to 1.06)
Age (years) at ART initiation			
0 to 2 years	1639	Ref.	Ref.
3 to 5 years		0.59 (0.33 to 1.04)	0.71 (0.39 to 1.30)
6 to 9 years		0.39 (0.22 to 0.68)	0.49 (0.27 to 0.89)
10 to 14 years		0.24 (0.09 to 0.63)	0.30 (0.11 to 0.82)
Time (years) on ART	1639	0.98 (0.92 to 1.04)	1.01 (0.93 to 1.10)
Caregiver vital status			
Mother or father deceased versus neither mother nor father deceased	1531	0.96 (0.73 to 1.25)	
Missing versus none		0.95 (0.75 to 1.19)	
On TB treatment at VL	1639	3.36 (1.01 to 11.22)	3.32 (1.13 to 9.81)
WHO stage at ART initiation			
Stage 3/4 & CDC Class B/C versus Stage 1/2 & Class A/N	1639	1.06 (0.85 to 1.32)	
Missing versus Stage 1/2 & Class A/N		1.13 (0.88 to 1.46)	
CD4 % at ART initiation >25 for children <5 years|CD4 count at ART initiation >350 for children aged >5 years			
Yes versus no	1612	0.77 (0.58 to 1.04)	0.76 (0.55 to 1.05)
Missing versus no		0.98 (0.79 to 1.22)	0.97 (0.76 to 1.24)
NNRTI base class at VL (vs. PI/other regimen)	1639	1.15 (0.77 to 1.72)	
ART regimen change for any reason before VL	1639	0.89 (0.57 to 1.38)	
Age at ART initiation × time (years) on ART			
0 to 2 years	1639	Ref.	Ref.
3 to 5 years		1.05 (0.95 to 1.15)	1.03 (0.93 to 1.14)
6 to 9 years		1.14 (1.01 to 1.28)	1.13 (1.00 to 1.28)
10 to 14 years		1.66 (1.14 to 2.40)	1.68 (1.16 to 2.43)

ART, antiretroviral therapy; ARV, antiretroviral; NNRTI, non‐nucleoside reverse transcriptase inhibitor; PI, protease inhibitor; TB, tuberculosis; VL, viral load; WHO, World Health Organization.

## 4 Discussion

In our study of children and their caregivers living with HIV, we found that children had more than twice the odds of not being virally suppressed if their caregivers were not virally suppressed, compared to children with suppressed caregivers. Caregivers experiencing an ART regimen change for any reason before the VL and receiving a non‐NNRTI‐based regimen at the time of the VL were both associated with viral non‐suppression in children. In Kenya, non‐NNRTI‐based regimens (e.g. protease or integrase inhibitor‐based regimens) typically constitute second‐ or third‐line ART for patients with HIV viraemia on first‐line, NNRTI‐based ART 27. Inadequate adherence is a common cause of HIV viraemia among adults receiving first‐ and second‐line ART in sub‐Saharan Africa, and adherence to first‐line ART has been shown to be a strong predictor of adherence to second‐line ART 42, 43, 44. Thus, although our study did not directly measure adherence, it is possible that experiencing an ART regimen change or being on a non‐NNRTI regimen are markers of prior inadequate adherence among caregivers that increased the risk of future inadequate adherence (i.e. at the time of the VL) for them and their children 42, 45. Young children are uniquely dependent on caregivers for their ART management, and it is plausible that caregivers’ adherence practices extended to their children, increasing the risk of viral non‐suppression for both individuals 9. Further research is needed to understand the patient and site‐level factors (e.g. availability of counselling and other interventions to enhance adherence) that contribute to viral non‐suppression among dyads, as well as the potential influence of adherence and drug resistance 46.

Overall viral suppression among caregivers and children in our study was suboptimal. The VL was suppressed for both the caregiver and child in only half of dyads, while 62% of children and 77% of caregivers were suppressed overall. These suppression estimates are lower than current national, facility‐based estimates for children and adults receiving ART in Kenya at 68% and 86%, respectively, underscoring the vulnerability of this population and the importance of understanding the barriers to viral suppression it experiences 47, 48. Achieving viral suppression for children in sub‐Saharan Africa is especially challenging. Studies have reported lower viral suppression rates for children and adolescents compared to adults in Kenya (57% to 66% vs. 63% to 87%) and in other low‐ and middle‐income countries (60% to 75% vs. 85%), as well as compared to children and adolescents in high‐income countries (≥ 90%) 49, 50, 51, 52, 53. A range of factors can influence adherence and viral suppression for children including the child's age, familial and socio‐economic environment, stigma, disclosure, and the physical and mental health status of children and caregivers 3, 10, 54, 55. Consistent with prior studies, we found that older child age was protective against viral non‐suppression compared to child age ≤2 years 56, 57, 58. This could reflect behavioural changes during childhood such as younger children refusing to take medications or the positive effects of HIV disclosure to older children, the effect of ART dosing frequency (i.e. twice daily dosing of lopinavir‐based ART in children <3 years vs. once daily efavirenz‐based ART for children ≥3 years and ≥35 kg), or biologic factors such as slower rates of viral suppression in infants compared to older children.

Additionally, children in our study who were receiving TB treatment at the time of the VL were three times more likely to not be virally suppressed compared to children not receiving TB treatment. This finding may be a statistical artefact given that only 0.8% of children were on TB treatment at the time of the VL (with this low percentage related at least in part the cross‐sectional nature of this variable among individuals enrolled in HIV care and on ART for ≥6 months). However, treatment for TB has been associated with a higher risk of viral non‐suppression in two studies of children living with HIV in South Africa, which may be due to the child receiving protease inhibitor‐based ART (e.g. pharmacologic interactions between ritonavir and rifampicin), inadequate ART adherence due to higher toxicities or pill burden, or biologic factors (e.g. immune activation in the setting of active TB causing HIV viraemia) 59, 60, 61, 62. Alternatively, the risk of incident TB may have been higher among children with worsening immune function due to ongoing viral replication, or among children with late‐onset immune reconstitution inflammatory syndrome following ART initiation 63.

Our findings suggest that a child's viral suppression status should be carefully assessed at the time his or her caregiver is found not to be virally suppressed. Some barriers to adherence and viral suppression may be common to both individuals, and a family‐centred management approach, rather than an individual approach, may be needed to address these barriers effectively. Differentiated care models that focus on the needs of families living with HIV are emerging 64, 65, 66, 67, 68, 69. In South Africa, family clubs are being used as ART distribution alternatives 64. In Uganda, implementing weekly clinics where children and adolescents were treated together with their families improved appointment adherence, and similar models have been implemented in urban centres in Kenya and through community groups in Namibia 65, 70. These care models may offer services that are better tailored to family issues and more efficient for programmes and patients 64, 71, 72, 73. To date, however, existing models have focused on clinically stable families and children with reliable caregivers, and knowledge gaps exist regarding models for at‐risk families 65, 72, 74, 75. Psychosocial interventions that address stigma, disclosure and mental health issues will likely play an important role in these models 3, 10, 54, 55, 76.

Our study has strengths and limitations. This study is, to our knowledge, the first to identify an association between viral suppression in children and their caregivers who were both living with HIV. Although others have investigated caregiver–child interactions that could influence adherence, our large cohort of caregiver–child dyads enabled us to directly evaluate the outcome of viral suppression 7, 8, 9, 77. The use of observational programme data carries limitations. Although the proportions of children included and excluded from the analysis were similar in terms of sex and age at ART initiation, subject selection may have been biased. Linking caregivers and children in the AMPATH medical record is an active process performed by clinicians or counsellors during routine care, and these individuals may have been less inclined to link non‐mother caregivers to their children in the medical record. We had limited insight into the roles caregivers played in their children's HIV care in our retrospective study (e.g. who was responsible for administering a child's medications), though understanding these roles is ultimately essential to understanding the meaning of the viral suppression association we identified. Despite this limited insight, it is likely that mothers, who comprised the large majority of caregivers in our study, were in fact responsible for their children's HIV management, which is consistent with other studies in sub‐Saharan Africa 5, 31. Finally, adherence and the factors associated with it are dynamic processes that can vary over time 78. We plan to examine the longitudinal nature of viral suppression among child–caregiver dyads in future research.

## 5 Conclusions

Among caregiver–child dyads in which both members were living with HIV, children were more likely to not be virally suppressed if their caregivers were not virally suppressed, compared to children with suppressed caregivers. A child's viral suppression status should be closely monitored if his or her caregiver is not virally suppressed.

## Competing interests

The authors declare that they have no competing interests.

## Authors’ contributions

J.M.H, E.A, B.L.G, A.G, J.W.H and K.W conceptualized and designed the study, and had primary responsibility for interpretation of the data. A.K and B.M analysed the data. J.M.H wrote the paper with assistance from E.A, B.G, A.G, J.W.H and K.K.W. All authors have read and approved the final manuscript.
